# Trajectories of Supportive Care Needs for People Who Travel to Receive Cancer Treatment: A Longitudinal Study in Australia

**DOI:** 10.1002/pon.70087

**Published:** 2025-02-10

**Authors:** Susannah K. Ayre, Elizabeth A. Johnston, Michael Ireland, Sonja March, Jeff Dunn, Suzanne Chambers, Belinda C. Goodwin

**Affiliations:** ^1^ Viertel Cancer Research Centre Cancer Council Queensland Fortitude Valley Australia; ^2^ School of Exercise and Nutrition Sciences Queensland University of Technology Kelvin Grove Australia; ^3^ Population Health Program QIMR Berghofer Medical Research Institute Herston Australia; ^4^ School of Psychology and Wellbeing University of Southern Queensland Ipswich Australia; ^5^ Centre for Health Research University of Southern Queensland Springfield Australia; ^6^ Faculty of Health Sciences Australian Catholic University Banyo Australia; ^7^ Exercise Medicine Research Institute Edith Cowan University Joondalup Australia; ^8^ School of Public Health and Social Work Queensland University of Technology Kelvin Grove Australia

**Keywords:** cancer, longitudinal, oncology, quality of life, supportive care, unmet needs

## Abstract

**Objective:**

To describe trajectories of change in unmet supportive care needs over a two‐year period among people diagnosed with cancer and assess whether these trajectories vary as a function of sociodemographic and clinical characteristics.

**Methods:**

This analysis used data from a longitudinal study of people in Queensland, Australia who travelled largely from regional and remote areas to metropolitan centres to receive cancer care (*N* = 784). Supportive care needs were measured at baseline, then at 3‐, 12‐, and 24‐month post‐baseline across five domains (‘psychological’, ‘physical and daily living’, ‘health systems and information’, ‘patient care and support’, ‘sexuality’) using the Supportive Care Needs Survey‐Short Form. Latent Curve Growth Analysis was performed to examine trajectories of change in unmet needs and assess whether these trajectories were influenced by participant characteristics.

**Results:**

Significant linear slopes indicated a modest decrease in unmet supportive care needs for all domains, except sexuality. For most domains, significant variance in intercepts but not slopes indicated individual differences in needs at baseline but not in trajectories over time. At baseline, the proportion of unmet needs was highest for the ‘physical and daily living’ (*M* = 44.2%, *SD* = 39.1%) and ‘psychological’ domains (*M* = 37.8%, *SD* = 36.3%). Unmet needs at baseline were consistently higher among participants who were younger, had a higher education level, and who reported poorer QoL.

**Conclusions:**

The proportion of unmet supportive care needs reported by people living with cancer may decrease over time, largely irrespective of sociodemographic and clinical characteristics. Despite this, unmet needs remain prevalent, particularly for physical and psychological support.

## 1 Background

Globally, an estimated 19.3 million people were diagnosed with cancer in 2020, with this number expected to increase to 28.4 million by 2040 [1]. Cancer and its treatment often result in multiple effects that can persist for years post‐diagnosis, impacting long‐term health, employment, and relationships [2]. Increasing recognition of the prolonged effects of cancer and its treatment has prompted research into the supportive care needs of cancer survivors [3]. ‘Supportive care needs’ refers to a multi‐dimensional construct, comprising self‐reported needs for informational, practical, physical, psychological, emotional, social, and spiritual support [4]. Having unmet needs for supportive care has been associated with adverse outcomes, including heightened psychological distress and reduced QoL [5].

To date, research exploring supportive care needs has been based primarily on cross‐sectional surveys or interviews with cancer survivors at a single stage of their disease trajectory [3]. A recent systematic review has demonstrated a high prevalence of unmet needs across various cancer types, particularly for physical and psychological support [6]. However, little is known about how supportive care needs change over time. Frameworks for optimal supportive care encompass all stages of the disease trajectory, from diagnosis, through treatment and survivorship, to end‐of‐life [7], meaning that longitudinal studies that examine trajectories of supportive care needs in cancer survivors over time are needed to inform effective service planning. With earlier detection and advancements in treatment leading to better survival [1], this planning is a priority to reduce the burden on increasingly resource‐constrained healthcare systems [8].

Compared to their urban counterparts, rural cancer survivors often report additional practical, psychosocial, and financial stressors due to their need to travel for treatment [9]. With 28% of Australians living outside major cities [10], it is vital that the supportive care needs of cancer survivors living in regional and remote Australia are better understood. Therefore, this longitudinal study aimed to i) describe trajectories of change in unmet supportive care needs over a two‐year period among a large sample of people who travelled to metropolitan cities to receive cancer treatment, and ii) assess whether these trajectories vary as a function of sociodemographic and clinical characteristics.

## 2 Methods

### 2.1 Participants and Procedure

This analysis used data from a longitudinal study of people staying at one of Cancer Council Queensland's subsidised accommodation lodges to receive cancer treatment in a nearby hospital between September 2017 and June 2020. Eligible participants were aged 18 years or older, able to read English, and living within the community (i.e., excluding hospital inpatients).

Data collection methods have been reported elsewhere [11]. Briefly, patients received an invitation to participate, either upon arrival at the lodge or via mail to their residential address following their stay at the lodge. Patients were contacted 1 week later via telephone to discuss the study and invited to mail back their completed consent form and questionnaire. Participants completed a self‐administered questionnaire at recruitment (baseline), and then at 3, 12, and 24 months. At recruitment, they also undertook a structured interview, either in‐person at the lodge or via telephone. Each interview and questionnaire required approximately 45 min of their time. Ethical approval was obtained from a recognised institutional Human Research Ethics Committee (reference no. H17REA152).

### 2.2 Measures

#### 2.2.1 Unmet Supportive Care Needs

Unmet supportive care needs were measured at all four timepoints using the Supportive Care Needs Survey‐Short Form (SCNS‐SF34) [12]. The SCNS‐SF34 is a 34‐item questionnaire that measures five domains of supportive care needs within the defined period of the past month [12]. Domains cover psychological (10 items; *α* ≥ 0.92), physical and daily living (5 items; *α* ≥ 0.85), health systems and information (11 items; *α* ≥ 0.94), patient care and support (5 items; *α* ≥ 0.87), and sexuality (3 items; *α* ≥ 0.85) needs. The questionnaire is a parsimonious measure of supportive care needs that has shown acceptable validity and reliability when used with cancer patients [12]. Participants responded to items using a five‐point scale. For participants who had completed most (≥ 50%) but not all items within a domain of the SCNS‐SF34 at any timepoint, missing item responses were replaced with the mean of all available item responses for that participant within the domain, rounded to the nearest whole number. Responses were then aggregated to form a binary variable for each item (0 = no or satisfied need; 1 = unmet need [low, moderate, or high]), thereby reflecting whether the need was present or not for each participant. Percentage scores were then calculated for each participant, reflecting the proportion of needs that remained unmet within each domain.

#### 2.2.2 Health‐Related QoL

Health‐related QoL was measured at all four timepoints using the EQ‐5D‐5L [13] as it has demonstrated adequate psychometric properties in diverse populations, including cancer patients [14]. It includes five items related to health, encompassing anxiety and depression, mobility, pain and discomfort, self‐care, and usual activities (e.g., work, study, housework, leisure activities; *α* ≥ 0.75) [13]. Items were scored on a five‐point scale ranging from 1 (‘no problems’) to 5 (‘extreme problems’), with higher scores indicating greater perceived severity in problems over the past day [13]. If participants had completed most (≥ 50%) but not all items within the EQ‐5D‐5L at any timepoint, missing item responses were replaced with the mean of all available item responses for that participant. Responses to the five items were then averaged to derive a single‐dimension score.

#### 2.2.3 Sociodemographic and Clinical Characteristics

At baseline, data were collected on age, gender, country of birth, native language, Indigenous status, highest level of education completed, relationship status, residential postcode, cancer type, time since diagnosis, presence of comorbidities, and access to private health insurance to cover cancer‐related treatment costs. Self‐reported cancer diagnoses were verified against the Queensland Cancer Register. Residential postcodes were used to determine Socio‐Economic Indexes for Areas (SEIFA) [15] and Accessibility/Remoteness Indexes of Australia (ARIA) [16] which serve as proxies for socioeconomic status and geographical remoteness, respectively.

### 2.3 Data Analysis

Participants with data for at least one domain of the SCNS‐SF34 at any timepoint were included in this analysis. For descriptive purposes, the proportion of participants reporting an unmet need for individual items on the SCNS‐SF34 was calculated for each timepoint. To visualise the domains with the highest proportion of unmet needs and changes over time, the mean proportion of unmet needs in each domain were plotted. To reduce skew, windorising was applied to the variable for time since diagnosis at baseline, whereby values exceeding 10 years were capped at 10 (*N* = 31). Additionally, in instances where data were collected before a confirmed diagnosis (resulting in negative values), values were adjusted to 0 (*N* = 3).

Initially, hierarchical clustering was performed on EQ‐5D‐5L items in SPSS v.29 [17] to determine whether trajectories of unmet needs differed according to QoL. This method revealed a two‐cluster solution (high vs. low QoL) across all timepoints post‐diagnosis (e.g., 3–6 months, 6–12 months). However, due to the small number of participants in the low QoL cluster at later timepoints (*N* < 10), trajectories of supportive care needs were not stratified by QoL in this analysis.

Latent Curve Growth Analysis (LCGA) using Mplus v.8.8 [18] was applied to examine the trajectory of change for each domain of supportive care needs over time. Additionally, factors that might influence the starting point (intercept) and rate of change (slope) of these trajectories were explored. The models were estimated by a full information maximum likelihood (FIML) method, enabling all available data to be used. Percentage scores tended to be positively skewed, particularly at later timepoints. To address this, a square root transformation was applied to the scores across all domains of supportive care needs for the LCGA.

Initial models for each domain incorporated both a latent intercept (i) and a latent linear slope (*s*). These values were determined by the average percentage domain score at baseline, and at 3, 12, and 24 months. These time scores were set at 0, 0.25, 1, and 2, mirroring baseline and the corresponding intervals. If data visualisation suggested a possible quadratic effect over time, a latent quadratic term (*q*) was introduced, and the model fit was compared with the linear model using AIC, BIC, and RMSEA statistics.

If either the intercept or slope had significant non‐zero residual variance, the following variables were introduced to the model to assess whether they explained residual variances: age (in years; mean centred), gender (male = 0 [ref]; female = 1), relationship status (not in a relationship = 0 [ref]; in a relationship = 1), education level (primary school = 0 [ref]; middle school = 1; secondary school = 2; tertiary education = 3), geographical remoteness (major city or inner regional area = 0 [ref]; outer regional or remote area = 1), socioeconomic status (percentile; mean centred), cancer type (6 dummy variables each comparing one cancer type against all others for breast, head and neck, lung, prostate, skin, and gynaecological cancer; other cancer type = 0 [ref]; specific cancer type = 1), time since diagnosis (years; mean centred), comorbidities (none = 0 [ref]; at least one = 1), private health insurance status (no access = 0 [ref]; access = 1), and health‐related QoL, all measured at baseline. For model simplicity, all covariates were treated as continuous. Except for QoL, all covariates were also considered time‐invariant. A separate LCGA was conducted to determine if QoL changed significantly over time. Since it remained constant, this variable at baseline was also treated as a time‐invariant covariate.

## 3 Results

### 3.1 Sample Characteristics

**FIGURE 1:**
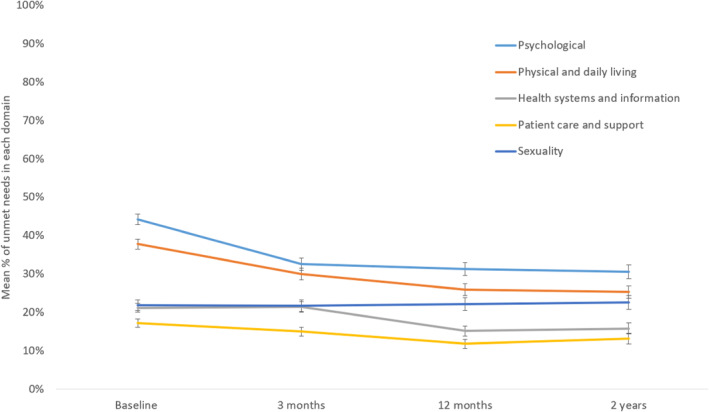
Mean proportion (%) of unmet needs for each supportive care needs domain over time with standard error bars.^†‡^^†^See Supporting Information S1: Table 2 for data. ^‡^Timepoints are in relation to baseline and do not indicate time since diagnosis.

Of the 811 people who consented to participate in the larger study, 784 (96.7%) were eligible for inclusion in this analysis. A flowchart of participant recruitment and inclusion is presented in Supporting Information S1: Figure 1. At baseline, participants were aged 26–92 years (*M* = 64.6, *SD* = 11.2) and 53.9% identified as male. Most participants were born in Australia (80.0%) and lived in outer regional or remote areas (52.1%). The sample was characterised by high socioeconomic disadvantage, with 51.8% residing in areas with the lowest three SEIFA deciles. Primary cancer diagnoses included breast (16.6%), head and neck (14.9%), skin (11.5%), and prostate (11.2%) cancers. Further sample characteristics are available in Supporting Information S1: Table 1.

At 24 months from baseline, 421 (53.7%) participants remained in the sample (see Supporting Information S1: Figure 1). While follow‐up surveys were distributed at 3‐, 12‐, and 24‐month post‐baseline, on average, they were returned after 4.2 (range = 1.8–13.1; *SD* = 1.0), 13.0 (range = 11.2–21.8; *SD* = 1.0), and 25.0 (range = 23.2–37.4; *SD* = 1.0) months, respectively. Several differences were observed between participants who completed the follow‐up surveys and those who did not. First, non‐Indigenous participants were more likely to complete the 24‐month survey compared to Indigenous participants (χ^2^_1_ = 3.86, *p* = 0.049). Similarly, participants who were in a relationship were more likely to complete the 12‐month (χ^2^_1_ = 6.94, *p* = 0.008) and 24‐month (χ^2^_1_ = 6.19, *p* = 0.013) surveys compared to participants who were not in a relationship. Additionally, participants with no comorbidities were more likely to complete the 24‐month survey compared to those with at least one comorbidity (χ^2^_1_ = 7.73, *p* = 0.005). The proportion of participants who completed the 24‐month survey also differed based on education level (χ^2^_4_ = 9.76, *p* = 0.045), with the highest proportion evident among participants who completed tertiary education (58.0%), and the lowest among those who completed primary school (43.0%). Similarly, participants with lung cancer were the least likely to complete the 3‐month (χ^2^_6_ = 24.02, *p* < 0.001), 12‐month (χ^2^_6_ = 31.11, *p* < 0.001), and 24‐month (χ^2^_6_ = 34.90, *p* < 0.001) surveys compared to other cancer types. Of the 784 participants, 207 (26.4%) withdrew due to medical reasons (e.g., deceased, receiving palliative care).

### 3.2 Unmet Supportive Care Needs Over Time

At baseline, the ‘physical and daily living’ domain showed the highest proportion of unmet needs (*M* = 44.2%, *SD* = 39.1%), followed by the ‘psychological’ domain (*M* = 37.8%, *SD* = 36.3%) (see Figure 1 and Supporting Information S1: Table 2). In the ‘physical and daily living’ domain, the most frequently reported unmet needs were for support with fatigue (49.9%) and difficulties completing usual activities (48.0%), whereas in the ‘psychological’ domain, fears about the cancer spreading (48.6%) and concerns about the emotional impact of cancer on family members and friends (48.2%) were the most prevalent unmet needs (see Supporting Information S1: Table 3). The proportion of unmet needs for support in the ‘health systems and information’ (*M* = 21.2%, *SD* = 32.0%) and ‘sexuality’ (*M* = 21.9%, *SD* = 36.1%) domains were similar, with unmet needs in the ‘patient care and support’ domain the lowest at baseline (*M* = 17.2%, *SD* = 30.5%). Significant linear slopes indicated a decrease in needs over time for all domains except ‘sexuality’ (see Figure 1 and Table 1).

**TABLE 1:** Latent curve growth analysis: Intercept and slope without covariates.

	Psychological	Physical and daily living	Health systems and information	Patient care and support	Sexuality
Linear models					
AIC	11060.890	11367.346	10847.670	10689.328	11267.320
BIC	11102.835	11409.245	10889.546	10731.227	11313.861
RMSEA	0.095	0.111	0.064	0.048	0.032
Intercept					
*Mean*	4.908*	5.227*	3.472*	2.897*	3.253*
(Variance)	(6.392*)	(5.923*)	(4.244*)	(3.284)	(5.817*)
Slope					
*Mean*	−0.426*	−0.306*	−0.352*	−0.241*	−0.053
(Variance)	0.339	(0.656)	0.367	(0.037)	(6.012*)
Quadratic models					
AIC		11381.494	10856.730	10697.263	
BIC		11423.393	10898.606	10739.162	
RMSEA		0.125	0.078	0.062	
Intercept					
*Mean*		5.109*	3.378*	2.827*	
(Variance)		(6.172*)	(4.315*)	(3.344*)	
Slope					
Mean		−0.091	−0.136*	(‐0.079*)	
(Variance)		0.275	(0.173)	0.014	

Abbreviations: AIC = akaike information criteria; BIC = bayesian information criteria; RMSEA = root mean square error of approximation.

*p < 0.05.

Fit statistics for quadratic models did not suggest better fit than the linear models, therefore covariates were tested in linear models only. Slope effect sizes from the linear models indicated stronger declines in unmet needs within the ‘psychological’, ‘health systems and information’, and ‘physical and daily living’ domains. Significant residual variance in the intercept of the ‘psychological’, ‘physical and daily living’, health systems and information’, and ‘sexuality’ domains was evident, suggesting significant individual differences in unmet needs at baseline across all domains, except ‘patient care and support’. Significant residual variance was also evident for the slope of the ‘sexuality’ domain, suggesting significant individual differences in the trajectory of these unmet needs. For the remaining domains, associations were therefore tested between the covariates and intercept only.

### 3.3 Covariates

Time since diagnosis, geographical remoteness, and access to private health insurance at baseline were not associated with the intercept or slope in any domain, indicating that these variables did not predict the proportion of unmet needs at baseline, nor their rate of change over the two‐year period (see Table 2). Age was negatively associated with the intercept across all domains, suggesting that older participants reported fewer unmet needs at baseline compared to younger participants. In contrast, education level was positively associated with the intercept across all domains, meaning that participants with higher education levels reported greater unmet needs compared to those with lower education levels. At baseline, female participants reported greater unmet needs than male participants in the ‘health systems and information’ domain, and participants in a relationship reported fewer unmet needs in the ‘sexuality’ domain compared to those not in a relationship. Additionally, participants living in higher socioeconomic status areas reported greater unmet needs in the ‘psychological’, ‘health systems and information’, and ‘patient care and support’ domains compared to those living in lower socioeconomic status areas.

**TABLE 2:** Latent curve growth analysis: Intercept and slope with covariates.

	Psychological	Physical and daily living	Health systems and information	Patient care and support	Sexuality
AIC	8661.563	8853.903	8664.987	8450.153	8999.065
BIC	8766.318	8958.616	8769.616	8554.824	9177.737
Intercept					
Mean	1.106	0.173	−0.024	0.231	2.539*
(Variance)	(3.668*)	(2.426*)	(2.668*)	(2.090*)	(4.047*)
Slope					
Mean	−0.374*	−0.254*	−0.330	−0.210*	−0.150
(Variance)	(0.034)	(1.180*)	(0.407)	(0.213)	(0.418)
*Covariates*	*Intercept*	*Intercept*	*Intercept*	*Intercept*	*Intercept/slope*
Age (years)	−0.574*	−0.246*	−0.209*	−0.203*	−0.765*/0.039
Gender^a^	0.436	0.392	0.619*	0.302	−0.318/−0.207
Relationship status^a^	−0.129	0.192	0.269	−0.043	−0.820*/−0.113
Education level^a^	0.214*	0.186*	0.327*	0.232*	0.250*/−0.053
Geographical remoteness^a^	0.008	−0.237	−0.012	−0.011	−0.174/0.054
Socioeconomic status	6.888*	6.553	8.481*	9.599*	4.112/1.467
Cancer type^b^					
Breast	−0.529	−0.187	−0.348	0.030	−0.045/0.578
Head and neck	−0.502	−0.352	−0.163	−0.071	−0.492/0.553
Prostate	−0.150	−0.711*	0.214	0.187	1.335*/0.225
Skin	−0.846*	−1.325*	−0.364	−0.281	−0.268/0.068
Lung	0.176	0.279	0.460	0.325	−0.011/0.862
Gynaecological	−0.566	−0.146	−0.472	0.130	−0.634/0.871*
Time since diagnosis (years)	−0.002	−0.053	−0.068	−0.035	−0.115/0.034
Comorbidities^a^	0.083*	0.543*	0.199	0.145	−0.323/0.159
PHI status^a^	0.293	−0.271	−0.075	−0.166	−0.286/0.067
QoL (baseline)	2.156*	2.709*	1.482*	1.450*	1.050*/0.150

Abbreviations: AIC = akaike information criteria; BIC = bayesian information criteria; PHI = private health insurance; QoL = quality of life.

^a^ Gender (male = 0 [ref]; female = 1); relationship status (not in a relationship = 0 [ref]; in a relationship = 1); education level (primary school = 0 [ref]; middle school = 1; secondary school = 2; tertiary education = 3); geographical remoteness (major city or inner regional area = 0 [ref]; outer regional or remote area = 1) comorbidities (none = 0 [ref]; at least one = 1); private health insurance status (no access = 0 [ref]; access = 1).

^b^ Cancer type was entered into the model as six dummy variables each comparing one cancer type against all others for breast, head and neck, lung, prostate, skin, and gynaecological cancer. For each variable, other cancer type = 0 [ref]; specific cancer type = 1.

*p < 0.05.

For the ‘physical and daily living’ domain, participants with skin and prostate cancer reported fewer unmet needs at baseline compared to those with other cancer types. Additionally, participants with skin cancer reported fewer unmet needs in the ‘psychological’ domain, while participants with prostate cancer reported greater unmet needs in the ‘sexuality’ domain. Perceived severity of QoL problems at baseline was also positively associated with unmet needs across all domains, and participants with at least one comorbidity reported greater unmet needs in the ‘psychological’ and ‘physical and daily living’ domains compared to those with no comorbidities. Finally, the significant variance in the slope for the ‘sexuality’ domain was associated with having gynaecological cancer compared to other cancer types. As shown in Supporting Information S1: Figure 2, participants with gynaecological cancer experienced a decrease in their mean proportion of unmet needs from baseline (*M* = 16.9, *SE* = 3.9) to 3 months (*M* = 14.3, *SE* = 4.8), followed by an increase that exceeded the baseline mean by 24 months (*M* = 20.7, *SE* = 5.4). Mean proportions of unmet sexuality needs across all four timepoints for each cancer type are reported in Supporting Information S1: Table 3.

## 4 Discussion

Across most domains of supportive care, this study demonstrated a modest but significant reduction in the level of unmet need reported by cancer survivors, largely living outside major cities, over a two‐year period. Managing fatigue, daily activities, fears about cancer progression or recurrence, and concerns about the emotional impact of cancer on loved ones were identified as key areas of unmet need, reported by almost half the sample at baseline. These results are consistent with a recent systematic review of supportive care needs in Australian cancer survivors [6], showing that unmet needs for psychological and physical support are prevalent, with weighted estimates on individual domain items ranging up to 47.1% and 42.8%, respectively, in studies on specific cancer types [6].

Although needs tended to decrease over time, a substantial proportion of participants in the current study still reported these needs two years later. Previous studies, largely focussed on one specific cancer type, have reported similar results, whereby needs tend to decrease over time [19, 20, 21, 22, 23, 24]. However, most studies only included one follow‐up assessment, typically within the first six months post‐diagnosis [19, 20, 22, 23, 24]. In the current study, where not all participants had been recently diagnosed with cancer at baseline, participants reported lower levels of unmet need across several domains at a 3‐month follow‐up, with little to no reduction in unmet needs over the next 21 months. This finding was irrespective of characteristics such as cancer type and time since diagnosis, demonstrating a generally reliable trend whereby people report fewer unmet needs when asked again after a short period of time. This result could reflect an improved ability to cope with and manage needs over time, including increased access to and use of support. Alternatively, the mere act of considering the need when prompted at baseline may have led to some participants seeking support, meaning that their need was satisfied by the subsequent survey. Further research is necessary to identify mechanisms underlying these results, and whether interventions prompting consideration of unmet needs could result in lower overall need.

Interestingly, despite the lack of access to local healthcare and support services, living in a more remote area was not associated with unmet needs in any domain; however, other sociodemographic and clinical factors were. Consistent with previous research [25], unmet needs in this study were generally higher among participants who were younger, attained a higher education level, or were living in a higher socioeconomic status area. Greater unmet needs in younger participants could be related to their need to return to usual routines, including employment or parenting responsibilities. In addition, participants with higher education levels or from higher socioeconomic status areas may have greater knowledge or awareness of their condition and expectations for their care. Subsequently, these participants may be more attuned to gaps or barriers in care they receive, reflected in higher domain scores. In the current study, female participants also had greater unmet needs for healthcare information and support compared to male participants. This finding aligns with previous research showing that female cancer survivors tend to seek support more frequently than their male counterparts, but are often less satisfied with the support received [26]. Additionally, unmet needs across several domains were greater among participants with at least one comorbidity or poorer QoL. Studies indicate that comorbidities may be associated with greater post‐operative complications and mortality among cancer survivors [27], which may result in heightened psychological and practical needs in this population group. Furthermore, there have been direct associations observed between poorer QoL and greater unmet needs [28]. Therefore, people with these characteristics may require routine early intervention for supportive care needs and regular follow‐up.

Unlike other domains, unmet sexuality needs did not significantly decrease over time, suggesting that needs relating to changes in sexual function and satisfaction tend to persist long‐term. This trend may reflect a lack of access or adherence to support for unmet sexuality needs, especially for men with prostate cancer or adults not in a relationship, who in the current study, reported greater unmet needs at baseline compared to their counterparts. For example, men with prostate cancer experience devastating impacts on sexual function but adherence to rehabilitation can be low, which may be partly due to the limited or delayed efficacy and side effects of medical interventions [29, 30]. Although prior studies have found that cancer survivors who are in a relationship tend to experience greater unmet needs in this domain [25], seeking support for sexual health may be a lower priority for those without a partner or spouse. In the current study, participants with gynaecological cancer also showed a distinct pattern in their sexuality needs over time, whereby the proportion of unmet needs decreased from baseline to the 3‐month follow‐up, before increasing thereafter. This finding indicates that while there may be an increased awareness and discussion among healthcare professionals and patients about the adverse effects of gynaecological cancer and its treatment on sexual health [31], the long‐term support for these needs may diminish over time. Even in cancer types that do not affect reproductive organs, perceived stigma or other priorities may prevent survivors from seeking support [32, 33], and clinicians' uncertainty about how to screen for and manage sexual dysfunction may contribute to sustained unmet needs [32, 33]. As the negative effects of cancer and its treatment on sexual health can adversely affect mood, self‐esteem, relationship adjustment, and overall QoL in cancer survivors [34], it is important to understand and address barriers to seeking or adhering to support.

### 4.1 Clinical Implications

Results from the current study have implications for the design and delivery of supportive care services. It was evident that a large proportion of cancer survivors experience unmet supportive care needs many years post‐diagnosis and initial treatment. As a priority, services are needed to provide support for managing fatigue, daily activities, fears of cancer progression or recurrence, concerns regarding the emotional impact of cancer on loved ones, and changes in sexual feelings and relationships. Healthcare professionals and community support organisations should be aware that although unmet needs may decrease over time, several needs remain prevalent. Significant individual differences in unmet needs at baseline also suggest that a case‐by‐case approach to care is warranted. Other studies should provide validation of these findings and explore additional factors that may explain individual differences in unmet needs. With more people living longer following a cancer diagnosis [1], and evidence of ongoing unmet needs in this population group, it is vital that services are cost‐effective, sustainable, and targeted towards priority needs [35]. Future research should explore solutions for optimising access to support, particularly in groups with higher unmet needs. Interventions that are both feasible and acceptable to the local context could be identified from prior research [36, 37] or co‐designed with participants [38], and trialled to determine their effectiveness in addressing unmet needs in cancer survivors across the care continuum.

### 4.2 Study Limitations

In the current study, attrition bias was evident with differences observed between the sociodemographic and clinical characteristics of participants who completed the follow‐up surveys and those who did not. Notably, participants who identified as Indigenous, attained a lower education level, were not in a relationship, lived with at least one comorbidity, and were diagnosed with lung cancer had higher attrition rates than other participants, limiting the capacity to draw conclusions around unmet needs in these groups. Additionally, people who were unable to read and/or speak English were ineligible to participate. As these characteristics have been associated with unmet supportive care needs in cancer patients [25, 39, 40], the generalisability of these findings to these groups may be limited. Despite their potential influence on supportive care needs, cancer stage and treatment modality were not included as covariates in the models due to a lack of reliable data on these variables in the current study. While the SCNS‐SF34 was used as a robust measurement tool in the current population [12], it does not capture several domains of unmet need, including financial, cognitive, and social needs. Additionally, items about treatment may not be applicable to individuals across all phases of survivorship. While this study provides important insights into the unmet supportive care needs of cancer survivors living in regional and remote areas, given the exploratory nature of this study, there is a potential risk of Type I errors, which underscores the need for further validation of these findings in future studies to draw more definitive conclusions.

## 5 Conclusions

This longitudinal study suggests that the proportion of unmet supportive care needs reported by people living with cancer may decrease over time, largely irrespective of sociodemographic and clinical characteristics. Despite this, unmet needs remain prevalent, particularly for physical and psychological support. An exception to this trend was for sexuality needs, whereby the average number of needs did not decrease significantly over the two‐year period. Baseline needs were consistently higher in participants who were younger, had a higher education level, and reported poorer QoL. To draw definitive conclusions regarding trajectories of supportive care needs over time, these findings should be validated through independent replication in other studies. With an increasing number of cancer survivors, it is vital that sustainable, cost‐effective, and targeted services are implemented early to address ongoing supportive care needs. Future research should aim to optimise access to support for these needs across the care continuum, particularly in priority populations.

## Author Contributions

B.C.G., S.M., J.D., and S.C. were involved in the study conception and design. B.C.G., M.I., and S.K.A. contributed to data analysis and interpretation. S.K.A., B.C.G., and E.A.J. wrote the first draft of the manuscript. All authors reviewed subsequent versions of the manuscript and have read and approved the final version.

## Conflicts of Interest

The authors declare no conflicts of interest.

## Supporting information

Supporting Information S1

## Data Availability

The data that support the findings of this study are available from the corresponding author upon reasonable request.

## References

[1] H. Sung , J. Ferlay , R. L. Siegel , et al., “Global Cancer Statistics 2020: GLOBOCAN Estimates of Incidence and Mortality Worldwide for 36 Cancers in 185 Countries,” CA: A Cancer Journal for Clinicians 71, no. 3 (2021): 209–249, 10.3322/caac.21660. 33538338

[2] C. Treanor and M. Donnelly , “Late Effects of Cancer and Cancer Treatment—The Perspective of the Patient,” Supportive Care in Cancer 24, no. 1 (2016): 337–346, 10.1007/s00520-015-2796-4. 26066051

[3] C. Paterson , K. Toohey , R. Bacon , P. S. Kavanagh , and C. Roberts , “What Are the Unmet Supportive Care Needs of People Affected by Cancer: An Umbrella Systematic Review,” Seminars in Oncology Nursing 39, no. 3 (2023): 151353, 10.1016/j.soncn.2022.151353. 36435657

[4] M. Fitch , “Supportive Care Framework,” Canadian Oncology Nursing Journal 18, no. 1 (2008): 6–14, 10.5737/1181912x181614. 18512565

[5] N. H. Hart , F. Crawford‐Williams , M. Crichton , et al., “Unmet Supportive Care Needs of People With Advanced Cancer and Their Caregivers: A Systematic Scoping Review,” Critical Reviews in Oncology 176 (2022): 103728, 10.1016/j.critrevonc.2022.103728. 35662585

[6] J. Roseleur , L. C. Edney , J. Jung , and J. Karnon , “Prevalence of Unmet Supportive Care Needs Reported by Individuals Ever Diagnosed With Cancer in Australia: A Systematic Review to Support Service Prioritisation,” Supportive Care in Cancer 31, no. 12 (2023): 676, 10.1007/s00520-023-08146-y. 37934313 PMC10630245

[7] M. Krishnasamy , A. Hyatt , H. Chung , K. Gough , and M. Fitch , “Refocusing Cancer Supportive Care: A Framework for Integrated Cancer Care,” Supportive Care in Cancer 31, no. 1 (2023): 14, 10.1007/s00520-022-07501-9. PMC9747818 36513841

[8] World Health Organization , Health Employment and Economic Growth: An Evidence Base (Geneva: World Health Organization, 2017), https://www.who.int/publications‐detail‐redirect/health‐employment‐and‐economic‐growth.

[9] P. N. Butow , F. Phillips , J. Schweder , K. White , C. Underhill , and D. Goldstein , “Psychosocial Well‐Being and Supportive Care Needs of Cancer Patients Living in Urban and Rural/regional Areas: A Systematic Review,” Supportive Care in Cancer 20 (2012): 1–22, 10.1007/s00520-011-1270-1. 21956760

[10] Australian Institute of Health and Welfare , Rural and Remote Health (Canberra: Australian Institute of Health and Welfare, 2023), https://www.aihw.gov.au/reports/rural‐remote‐australians/rural‐and‐remote‐health.

[11] E. A. Johnston , N. Craig , A. Stiller , et al., “The Impact on Employment for Rural Cancer Patients and Their Caregivers Travelling to Major Cities for Treatment,” Health and Social Care in the Community 2023 (2023): e6728504, 10.1155/2023/6728504.

[12] A. Boyes , A. Girgis , and C. Lecathelinais , “Brief Assessment of Adult Cancer Patients’ Perceived Needs: Development and Validation of the 34‐item Supportive Care Needs Survey (SCNS‐SF34),” Journal of Evaluation in Clinical Practice 15, no. 4 (2009): 602–606, 10.1111/j.1365-2753.2008.01057.x. 19522727

[13] M. Herdman , C. Gudex , A. Lloyd , et al., “Development and Preliminary Testing of the New Five‐Level Version of EQ‐5D (EQ‐5D‐5L),” Quality of Life Research 20, no. 10 (2011): 1727–1736, 10.1007/s11136-011-9903-x. 21479777 PMC3220807

[14] Y.‐S. Feng , T. Kohlmann , M. F. Janssen , and I. Buchholz , “Psychometric Properties of the EQ‐5D‐5L: A Systematic Review of the Literature,” Quality of Life Research 30, no. 3 (2021): 647–673, 10.1007/s11136-020-02688-y. 33284428 PMC7952346

[15] Australian Bureau of Statistics , Census of Population and Housing: Socio‐Economic Indexes for Areas (SEIFA) (Canberra: Commonwealth of Australia, 2016), https://www.abs.gov.au/ausstats/abs@.nsf/Lookup/by%20Subject/2033.0.55.001~2016~Main%20Features~IRSD~19.

[16] Australian Bureau of Statistics , Remoteness Areas (Canberra: Commonwealth of Australia, 2023), https://www.abs.gov.au/statistics/standards/australian‐statistical‐geography‐standard‐asgs‐edition‐3/jul2021‐jun2026/remoteness‐structure/remoteness‐areas.

[17] IBM Corp , IBM SPSS Statistics for Windows (Chicago: SPSS Inc).

[18] L. K. Muthén and B. O. Muthén , Mplus User’s Guide (Los Angeles, CA: Muthén & Muthén, 1998).

[19] M. Minstrell , T. Winzenberg , N. Rankin , C. Hughes , and J. Walker , “Supportive Care of Rural Women With Breast Cancer in Tasmania, Australia: Changing Needs over Time,” Psycho‐Oncology 17, no. 1 (2008): 58–65, 10.1002/pon.1174. 17410518

[20] M. E. McDowell , S. Occhipinti , M. Ferguson , J. Dunn , and S. K. Chambers , “Predictors of Change in Unmet Supportive Care Needs in Cancer,” Psycho‐Oncology 19, no. 5 (2010): 508–516, 10.1002/pon.1604. 19598292

[21] V. L. Beesley , M. A. Price , P. M. Webb , P. O'Rourke , L. Marquart , and P. N. Butow , “Changes in Supportive Care Needs After First‐Line Treatment for Ovarian Cancer: Identifying Care Priorities and Risk Factors for Future Unmet Needs,” Psycho‐Oncology 22, no. 7 (2013): 1565–1571, 10.1002/pon.3169. 22936668

[22] D. Langbecker and P. Yates , “Primary Brain Tumor Patients’ Supportive Care Needs and Multidisciplinary Rehabilitation, Community and Psychosocial Support Services: Awareness, Referral and Utilization,” Journal of Neuro‐Oncology 127, no. 1 (2016): 91–102, 10.1007/s11060-015-2013-9. 26643806

[23] P. C. Valery , C. M. Bernardes , V. Beesley , A. L. Hawkes , P. Baade , and G. Garvey , “Unmet Supportive Care Needs of Australian Aboriginal and Torres Strait Islanders With Cancer: A Prospective, Longitudinal Study,” Supportive Care in Cancer 25, no. 3 (2017): 869–877, 10.1007/s00520-016-3475-9. 27834004

[24] K. Barr , D. Hill , A. Farrelly , M. Pitcher , and V. White , “Unmet Information Needs Predict Anxiety in Early Survivorship in Young Women With Breast Cancer,” J. Cancer Surviv. 14, no. 6 (2020): 826–833, 10.1007/s11764-020-00895-7. 32514909

[25] P. T. Okediji , O. Salako , and O. O. Fatiregun , “Pattern and Predictors of Unmet Supportive Care Needs in Cancer Patients,” Cureus 9 (2017): e1234, 10.7759/cureus.1234. 28620565 PMC5467772

[26] S.‐A. Clarke , L. Booth , G. Velikova , and J. Hewison , “Social Support: Gender Differences in Cancer Patients in the United Kingdom,” Cancer Nursing 29, no. 1 (2006): 66–72, 10.1097/00002820-200601000-00012. 16557124

[27] M. Søgaard , R. W. Thomsen , K. S. Bossen , H. T. Sørensen , and M. Nørgaard , “The Impact of Comorbidity on Cancer Survival: A Review,” Clinical Epidemiology 5 (2013): 3–29, 10.2147/CLEP.S47150. 24227920 PMC3820483

[28] D. G. Hansen , P. V. Larsen , L. V. Holm , N. Rottmann , S. H. Bergholdt , and J. Søndergaard , “Association Between Unmet Needs and Quality of Life of Cancer Patients: A Population‐Based Study,” Acta Oncologica 52, no. 2 (2013): 391–399, 10.3109/0284186x.2012.742204. 23244672

[29] S. K. Chambers , S. Occhipinti , L. Schover , et al., “A Randomised Controlled Trial of a Couples‐Based Sexuality Intervention for Men With Localised Prostate Cancer and Their Female Partners,” Psycho‐Oncology 24, no. 7 (2015): 748–756, 10.1002/pon.3726. 25483780

[30] S. K. Chambers , S. Occhipinti , A. Stiller , et al., “Five‐Year Outcomes From a Randomised Controlled Trial of a Couples‐Based Intervention for Men With Localised Prostate Cancer,” Psycho‐Oncology 28, no. 4 (2019): 775–783, 10.1002/pon.5019. 30716188

[31] A. Ben Charif , A. D. Bouhnik , B. Courbiere , et al., “Patient Discussion About Sexual Health With Health Care Providers After Cancer‐A National Survey,” Journal of Sexual Medicine 13, no. 11 (2016): 1686–1694, 10.1016/j.jsxm.2016.09.005. 27686697

[32] Y. Dai , O. Y. Cook , L. Yeganeh , C. Huang , J. Ding , and C. E. Johnson , “Patient‐reported Barriers and Facilitators to Seeking and Accessing Support in Gynecologic and Breast Cancer Survivors With Sexual Problems: A Systematic Review of Qualitative and Quantitative Studies,” Journal of Sexual Medicine 17, no. 7 (2020): 1326–1358, 10.1016/j.jsxm.2020.03.004. 32331967

[33] A. Zhu and D. Wittmann , “Barriers to Sexual Recovery in Men With Prostate, Bladder and Colorectal Cancer,” Urologic Oncology: Seminars and Original Investigations 40, no. 9 (2022): 395–402, 10.1016/j.urolonc.2020.08.005. 32868190

[34] J. M. Ussher , J. Perz , and E. Gilbert , and The Australian Cancer and Sexuality Study Team , “Perceived Causes and Consequences of Sexual Changes After Cancer for Women and Men: A Mixed Method Study,” BMC Cancer 15, no. 1 (2015): 268, 10.1186/s12885-015-1243-8. 25885443 PMC4407322

[35] M. Jefford , D. Howell , Q. Li , et al., “Improved Models of Care for Cancer Survivors,” Lancet 399, no. 10334 (2022): 1551–1560, 10.1016/s0140-6736(22)00306-3. 35430022 PMC9009839

[36] L. C. Edney , J. Roseleur , J. Gray , B. Koczwara , and J. Karnon , “Mapping a Decade of Interventions to Address the Supportive Care Needs of Individuals Living With or beyond Cancer: A Scoping Review of Reviews,” Supportive Care in Cancer 30, no. 5 (2022): 3793–3804, 10.1007/s00520-021-06713-9. 35029770

[37] B. C. Goodwin , L. Zajdlewicz , A. Stiller , et al., “What Are the Post‐treatment Information Needs of Rural Cancer Survivors in Australia? A Systematic Literature Review,” Psycho‐Oncology 32, no. 7 (2023): 1001–1012, 10.1002/pon.6169. 37248643

[38] T. Green , A. Bonner , L. Teleni , et al., “Use and Reporting of Experience‐Based Codesign Studies in the Healthcare Setting: A Systematic Review,” BMJ Quality and Safety 29, no. 1 (2020): 64–76, 10.1136/bmjqs-2019-009570. 31548278

[39] C. M. Bernardes , V. Beesley , J. Martin , et al., “Unmet Supportive Care Needs Among People With Cancer: A Cross‐Cultural Comparison Between Indigenous and Non‐indigenous Australians,” European Journal of Cancer Care 28, no. 5 (2019): e13080, 10.1111/ecc.13080. 31094021

[40] Š. Miroševič , J. B. Prins , P. Selič , L. Zaletel Kragelj , and Z. Klemenc Ketiš , “Prevalence and Factors Associated With Unmet Needs in Post‐treatment Cancer Survivors: A Systematic Review,” European Journal of Cancer Care 28, no. 3 (2019): e13060, 10.1111/ecc.13060. 31008544

