# Supplementary material for: Trajectories of Supportive Care Needs for People Who Travel to Receive Cancer Treatment: A Longitudinal Study in Australia
Source: Psychooncology. 2025 Feb 10;34(2):e70087. doi: 10.1002/pon.70087 (PMC11811482; doi:10.1002/pon.70087)
Supplement: Supplementary file 1 — Supporting Information S1 [file PON-34-e70087-s001.docx]

**
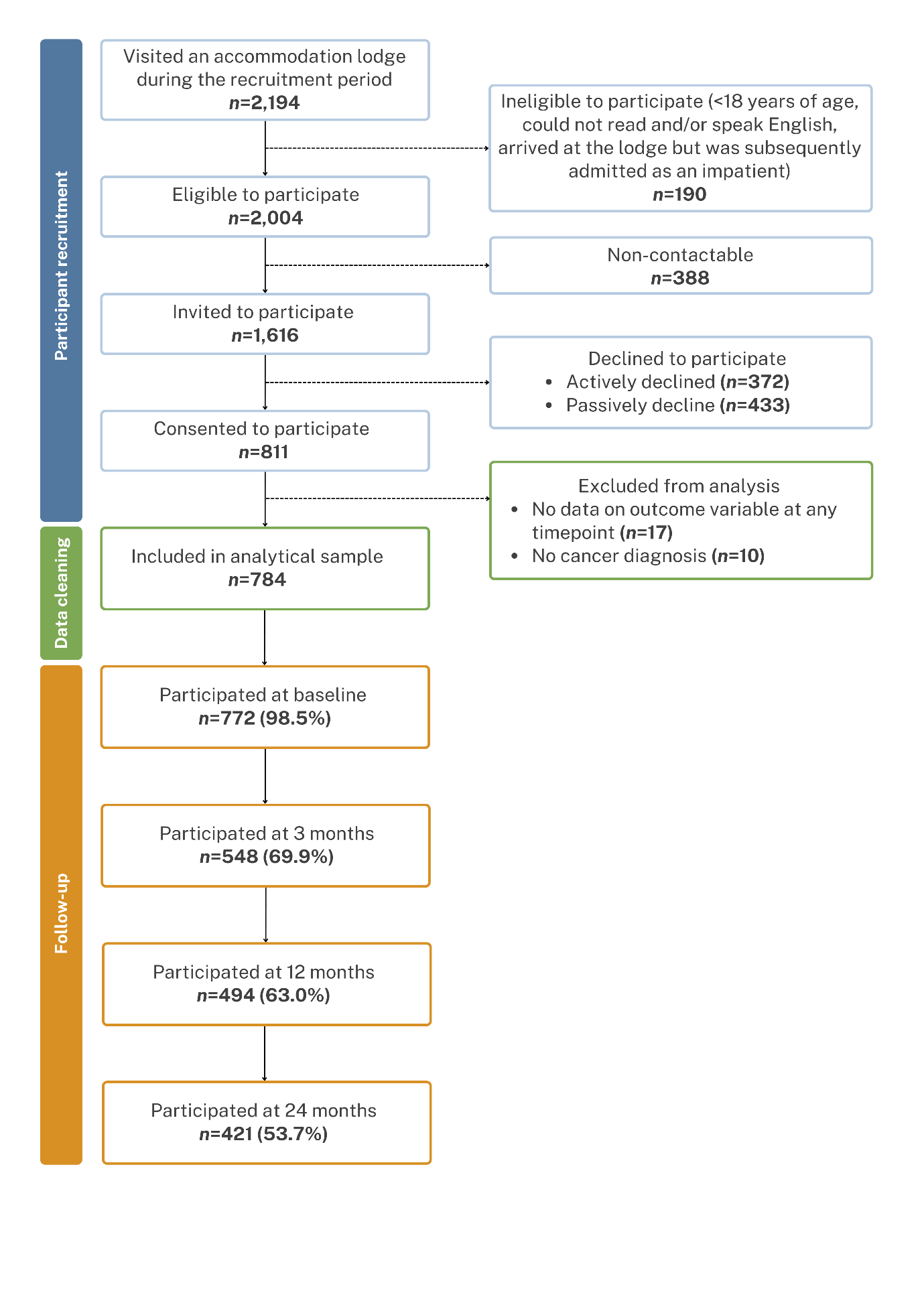
**

**Supplementary Figure 1.** Flowchart of participants through the study.

**Supplementary Table 1.** Sociodemographic and clinical characteristics of the sample (*n*=784).

|  | Results |
| --- | --- |
| Sociodemographic characteristics at baseline | |
| Age (years), mean (standard deviation)† | 64.6 (11.2) |
| Gender, *n* (%)‡ |  |
| Female | 359 (46.1) |
| Male | 419 (53.9) |
| Country of birth, *n* (%)‡ |  |
| Australia | 545 (80.0) |
| Other | 136 (20.0) |
| Native language, *n* (%)‡ |  |
| English | 660 (96.9) |
| Other | 21 (3.1) |
| Indigenous status, *n* (%)‡ |  |
| Yes | 33 (4.4) |
| No | 717 (95.6) |
| Highest level of education completed, *n* (%)‡ |  |
| Primary school (Year 9 or below) | 107 (14.2) |
| Middle school (Year 10) | 233 (30.8) |
| Secondary school (Year 12) | 109 (14.4) |
| Tertiary education | 307 (40.6) |
| Relationship status, *n* (%)‡ |  |
| In a relationship (married, de facto, partnered) | 503 (66.2) |
| Not in a relationship (single, divorced, or widowed) | 257 (33.8) |
| Socioeconomic status, *n* (%)‡ |  |
| High (deciles 7-10) | 89 (11.4) |
| Medium (deciles 4-6) | 287 (36.8) |
| Low (deciles 1-3) | 404 (51.8) |
| Geographical remoteness, *n* (%)‡ |  |
| Major city and inner regional | 374 (47.9) |
| Outer regional and remote | 406 (52.1) |
| Clinical characteristics at baseline |  |
| Cancer type, *n* (%)‡ |  |
| Breast | 130 (16.6) |
| Head and neck | 117 (14.9) |
| Skin | 90 (11.5) |
| Prostate | 88 (11.2) |
| Gynaecological | 68 (8.7) |
| Lung | 53 (6.8) |
| Other | 238 (30.4) |
| Time since diagnosis (years), median (interquartile range)§ | 0.5 (0.3-1.8) |
| Access to private health insurance to cover cancer-related treatment costs, *n* (%)‡ |  |
| Yes | 136 (21.0) |
| No | 511 (79.0) |
| Presence of comorbidities, *n* (%)‡ |  |
| None | 272 (42.5%) |
| One or more | 368 (57.5%) |
| Health-related quality of life, median (interquartile range)¶ | 1.6 (1.2-2.0) |

† Missing data for 6 (0.8%) cases.

‡ Valid percentage calculated from sample (excluding missing or unknown data).

§ Missing data for 46 (5.9%) cases.

¶ Measured using the EQ-5D-5L. Missing data for 5 (0.6%) cases.

**Supplementary Table 2.** Mean proportion of unmet needs within each supportive care needs domain on the SCNS-SF34.†

|  | Mean (standard deviation) proportion of unmet needs | | | |
| --- | --- | --- | --- | --- |
|  | Baseline | 3 months | 12 months | 24 months |
|  | (*n*=750-765)‡ | *(n*=535-548)‡ | (*n*=483-493)‡ | (*n*=410-421)‡ |
| Supportive care needs domain |  |  |  |  |
| Health systems and information | 21.2 (32.0) | 21.5 (32.4) | 15.2 (28.6) | 15.8 (30.6) |
| Patient care and support | 17.2 (30.5) | 15.0 (26.5) | 11.8 (26.9) | 13.1 (28.6) |
| Physical and daily living | 44.2 (39.1) | 32.6 (37.2) | 31.3 (36.8) | 30.6 (36.2) |
| Psychological | 37.8 (36.3) | 30.0 (34.4) | 26.0 (33.8) | 25.3 (33.3) |
| Sexuality | 21.9 (36.1) | 21.7 (36.2) | 22.2 (36.4) | 22.6 (37.0) |

† SCNS-SF34= Supportive Care Needs Short Form-34.
‡ The sample size varied across different domains due to missing data. Participants were excluded from the respective models if data were missing on all or most items (i.e., >50% items) within the supportive care needs domain.

**Supplementary Table 3.** Number and proportion (%) of the sample reporting an unmet need on individual items within the SCNS-SF34.†

|  | Number and proportion (%) of participants reporting an unmet need‡§ | | | |
| --- | --- | --- | --- | --- |
|  | Baseline | 3 months | 12 months | 24 months |
| Health systems and information | *n*=757 | *n*=544 | *n*=489 | *n*=417 |
| Being given written information about the important aspects of your care | 150 (19.8) | 114 (21.0) | 76 (15.5) | 62 (14.9) |
| Being given information (written, diagrams, drawings) about aspects of managing your illness and side-effects at home | 170 (22.5) | 108 (19.9) | 81 (16.6) | 74 (17.7) |
| Being given explanations of those tests for which you would like explanations | 160 (21.1) | 125 (23.0) | 76 (15.5) | 69 (16.5) |
| Being adequately informed about the benefits and side-effects of treatments before you choose to have them | 155 (20.5) | 126 (23.2) | 78 (16.0) | 69 (16.5) |
| Being informed about your test results as soon as feasible | 182 (24.0) | 135 (24.8) | 78 (16.0) | 69 (16.5) |
| Being informed about cancer which is under control or diminishing | 175 (23.1) | 140 (25.7) | 76 (15.5) | 61 (14.6) |
| Being informed about things you can do to help yourself to get well | 183 (24.2) | 125 (23.0) | 80 (16.4) | 73 (17.5) |
| Having access to professional counselling (e.g., psychologist, social worker, counsellor, nurse specialist) if you, family, or friends need it | 182 (24.0) | 112 (20.6) | 79 (16.2) | 71 (17.0) |
| Being treated like a person, not just another case | 126 (16.6) | 103 (18.9) | 74 (15.1) | 60 (14.4) |
| Being treated in a hospital or clinic that is as physically pleasant as possible | 115 (15.2) | 88 (16.2) | 43 (8.8) | 50 (12.0) |
| Having one member of hospital staff with whom you can talk to about all aspects of your condition, treatment, and follow-up | 167 (22.1) | 110 (20.2) | 75 (15.3) | 68 (16.3) |
| Patient care and support | *n*=763 | *n*=546 | *n*=490 | *n*=418 |
| More choice about which cancer specialists you see | 145 (19.0) | 84 (15.4) | 69 (14.1) | 67 (16.0) |
| More choice about which hospital you attend | 131 (17.2) | 72 (13.2) | 61 (12.4) | 53 (12.7) |
| Reassurance by medical staff that the way you feel is normal | 143 (18.7) | 100 (18.3) | 63 (12.9) | 58 (13.9) |
| Hospital staff attending promptly to your physical needs | 115 (15.1) | 73 (13.4) | 52 (10.6) | 51 (12.2) |
| Hospital staff acknowledging, and showing sensitivity to, your feelings and emotional needs | 123 (16.1) | 80 (14.7) | 44 (9.0) | 45 (10.8) |
| Physical and daily living | *n*=750 | *n*=547 | *n*=490 | *n*=421 |
| Pain | 321 (42.8) | 173 (31.6) | 169 (34.5) | 140 (33.3) |
| Lack of energy/tiredness | 374 (49.9) | 213 (38.9) | 182 (37.1) | 151 (35.9) |
| Feeling unwell a lot of the time | 282 (37.6) | 138 (25.2) | 120 (24.5) | 91 (21.6) |
| Work around the home | 322 (42.9) | 160 (29.3) | 132 (26.9) | 110 (26.1) |
| Not being able to do the things you used to do | 360 (48.0) | 207 (37.8) | 163 (33.3) | 152 (36.1) |
| Psychological | *n*=765 | *n*=548 | *n*=493 | *n*=421 |
| Anxiety | 262 (34.2) | 128 (23.4) | 110 (22.3) | 84 (20.0) |
| Feeling down or depressed | 245 (32.0) | 142 (25.9) | 125 (25.4) | 99 (23.5) |
| Feelings of sadness | 242 (31.6) | 138 (25.2) | 108 (21.9) | 96 (22.8) |
| Fears about the cancer spreading | 372 (48.6) | 230 (42.0) | 184 (37.3) | 148 (35.2) |
| Worry that the results of treatment are beyond your control | 343 (44.8) | 182 (33.2) | 146 (29.6) | 116 (27.6) |
| Uncertainty about the future | 350 (45.8) | 208 (38.0) | 158 (32.0) | 127 (30.2) |
| Learning to feel in control of your situation | 266 (34.8) | 156 (28.5) | 112 (22.7) | 91 (21.6) |
| Keeping a positive outlook | 233 (30.5) | 135 (24.6) | 95 (19.3) | 89 (21.1) |
| Feelings about death and dying | 210 (27.5) | 131 (23.9) | 106 (21.5) | 83 (19.7) |
| Concerns about the worries of those close to you | 369 (48.2) | 194 (35.4) | 137 (27.8) | 132 (31.4) |
| Sexuality | *n*=753 | *n*=535 | *n*=483 | *n*=410 |
| Changes in your sexual feelings | 188 (25.0) | 141 (26.4) | 132 (27.3) | 111 (27.1) |
| Changes in your sexual relationships | 174 (23.1) | 130 (24.3) | 119 (24.6) | 99 (24.1) |
| Being given information about sexual relationships | 132 (17.5) | 78 (14.6) | 71 (14.7) | 68 (16.6) |

† SCNS-SF34= Supportive Care Needs Short Form-34.

‡ Valid percentage calculated from sample excluding missing data.

§ For missing item responses that were substituted with the mean of the available item responses within that domain, values were rounded to the nearest whole number.

**Supplementary Figure 2.** Trajectory of sexuality needs for participants with gynaecological cancer compared to all other cancer types (with means and standard errors).‡


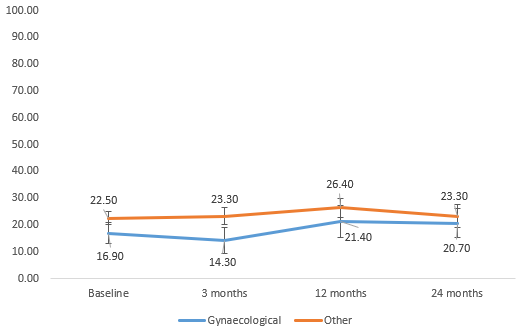


‡Timepoints are in relation to baseline and do not indicate time since diagnosis.

**Supplementary Table 4.** Mean proportion of unmet sexuality needs on the SCNS-SF34† by cancer type.

|  | **Mean (standard error) proportion of unmet needs** | | | |
| --- | --- | --- | --- | --- |
|  | **Baseline** | **3 months** | **12 months** | **24 months** |
| **Health systems and information needs** | | | | |
| All | 21.2 (1.2) | 21.5 (1.4) | 15.2 (1.3) | 15.8 (1.5) |
| Breast | 16.8 (2.5) | 23.6 (3.3) | 16.6 (2.9) | 19.3 (3.6) |
| Head and neck | 20.0 (3.0) | 15.9 (2.8) | 13.8 (3.1) | 10.6 (3.0) |
| Skin | 21.7 (3.7) | 21.0 (4.0) | 7.4 (2.2) | 7.8 (2.8) |
| Prostate | 19.0 (3.4) | 14.6 (3.3) | 10.3 (2.8) | 12.8 (3.4) |
| Gynaecological | 18.8 (3.7) | 20.3 (4.6) | 17.3 (4.2) | 23.0 (6.0) |
| Lung | 30.8 (5.3) | 27.6 (7.1) | 27.3 (7.5) | 35.5 (9.0) |
| Other | 23.3 (2.2) | 26.7 (3.0) | 18.9 (3.2) | 16.4 (3.4) |
| **Patient care and support needs** | | | | |
| All | 17.2 (1.1) | 15.0 (1.1) | 11.8 (1.2) | 13.1 (1.4) |
| Breast | 15.3 (2.5) | 15.2 (2.8) | 13.5 (2.9) | 15.0 (3.3) |
| Head and neck | 15.1 (2.7) | 10.9 (2.4) | 11.0 (3.0) | 10.1 (3.0) |
| Skin | 16.8 (3.5) | 14.0 (3.3) | 6.4 (2.1) | 8.2 (3.1) |
| Prostate | 16.0 (3.3) | 10.1 (2.7) | 7.5 (2.6) | 8.7 (2.9) |
| Gynaecological | 14.7 (3.3) | 19.1 (4.2) | 13.8 (4.2) | 18.9 (5.5) |
| Lung | 24.2 (4.9) | 18.1 (5.6) | 20.0 (6.6) | 22.9 (7.6) |
| Other | 19.1 (2.1) | 18.6 (2.3) | 14.1 (2.9) | 15.2 (3.4) |
| **Physical and daily living needs** | | | | |
| All | 44.2 (1.4) | 32.6 (1.6) | 31.3 (1.7) | 30.6 (1.8) |
| Breast | 42.0 (3.5) | 34.5 (3.7) | 32.2 (3.9) | 31.4 (4.1) |
| Head and neck | 34.8 (3.4) | 30.6 (3.8) | 24.6 (3.8) | 28.1 (4.1) |
| Skin | 35.4 (4.1) | 23.4 (4.3) | 21.5 (4.1) | 20.4 (3.9) |
| Prostate | 31.4 (3.8) | 20.0 (3.8) | 19.7 (3.6) | 24.5 (4.3) |
| Gynaecological | 57.2 (4.8) | 35.4 (5.6) | 41.9 (59) | 35.7 (6.2) |
| Lung | 51.7 (5.6) | 49.7 (6.9) | 46.2 (7.9) | 45.7 (8.2) |
| Other | 52.8 (2.6) | 38.8 (3.2) | 40.7 (3.6) | 36.9 (4.1) |
| **Psychological needs** | | | | |
| All | 37.8 (1.3) | 30.0 (1.5) | 26.0 (1.5) | 25.3 (1.6) |
| Breast | 34.2 (3.3) | 31.1 (3.4) | 28.1 (3.6) | 27.4 (3.8) |
| Head and neck | 33.2 (3.2) | 27.2 (3.5) | 21.9 (3.2) | 18.5 (3.4) |
| Skin | 34.7 (3.7) | 22.9 (3.9) | 17.3 (3.6) | 19.6 (3.9) |
| Prostate | 30.7 (3.7) | 22.4 (3.5) | 15.2 (3.1) | 19.5 (3.6) |
| Gynaecological | 39.1 (4.5) | 29.6 (4.7) | 27.9 (5.4) | 29.2 (5.1) |
| Lung | 43.4 (5.4) | 38.8 (6.8) | 42.3 (8.4) | 42.4 (9.2) |
| Other | 44.2 (2.4) | 36.9 (3.2) | 34.2 (3.5) | 30.8 (3.9) |
| **Sexuality needs** | | | | |
| All | 21.9 (1.3) | 21.7 (1.6) | 22.2 (1.7) | 22.6 (1.8) |
| Breast | 22.3 (3.4) | 23.5 (3.7) | 22.9 (3.8) | 22.8 (4.2) |
| Head and neck | 16.2 (3.0) | 19.4 (3.6) | 18.3 (3.6) | 21.6 (4.4) |
| Skin | 20.6 (3.9) | 17.2 (3.7) | 9.7 (3.4) | 12.7 (3.6) |
| Prostate | 32.5 (4.3) | 30.5 (5.0) | 28.8 (4.9) | 31.1 (5.3) |
| Gynaecological | 16.9 (3.9) | 14.3 (4.8) | 21.4 (5.9) | 20.7 (5.4) |
| Lung | 22.0 (5.5) | 16.7 (5.7) | 30.6 (8.7) | 28.3 (8.8) |
| Other | 22.5 (2.4) | 23.3 (3.3) | 26.4 (3.6) | 23.3 (4.2) |

† SCNS-SF34= Supportive Care Needs Short Form-34.
